# Catalytic Performance of a New 1D Cu(II) Coordination Polymer {Cu(NO_3_)(H_2_O)}(HTae)(4,4′-Bpy) for Knoevenagel Condensation

**DOI:** 10.3390/molecules21121651

**Published:** 2016-12-01

**Authors:** Edurne S. Larrea, Roberto Fernández de Luis, María I. Arriortua

**Affiliations:** 1 Department Mineralogía y Petrología, Universidad del País Vasco, UPV/EHU, Sarriena s/n, 48940 Leioa, Spain; maribel.arriortua@ehu.eus; 2 BCMaterials (Basque Center for Materials, Applications & Nanostructures), Technological Park of Zamudio, Camino de Ibaizabal, Bndg. 500-1st, 48160 Derio, Spain; roberto.fernandez@bcmaterials.net

**Keywords:** coordination polymer, heterogeneous catalysis, Knoevenagel condensation

## Abstract

The {Cu(NO_3_)(H_2_O)}(HTae)(4,4′-Bpy) (H_2_Tae = 1,1,2,2-tetraacetylethane, 4,4′-Bpy = 4,4′-Dipyridyl) 1D coordination polymer has been obtained by slow evaporation. The crystal structure consists of parallel and oblique {Cu(HTae)(4,4′-Bpy)} zig-zag metal–organic chains stacked along the [100] crystallographic direction. Copper(II) ions are in octahedral coordination environment linked to two nitrogen atoms of two bridging 4,4′-Bpy and to two oxygen atoms of one HTae molecule in the equatorial plane. The occupation of the axial positions varies from one copper atom to another, with different combinations of water molecules and nitrate anions, giving rise to a commensurate super-structure. By means of the thermal removal of water molecules, copper coordinatively unsaturated centres are obtained. These open metal sites could act as Lewis acid active sites in several heterogeneous catalytic reactions. The dehydrated compound, **CuHTaeBpy_HT**, has been tested as a heterogeneous recoverable catalyst for Knoevenagel condensation reactions. The catalyst is active and heterogeneous for the condensation of aldehydes with malononitrile at 60 °C using a molar ratio catalyst:substrate of 3 % and toluene as solvent. The catalyst suffers a partial loss of activity when reusing it, but can be reused at least four times.

## 1. Introduction

The research made on coordination polymers, or metal–organic frameworks (MOFs), during the last decades is very impressive due to the potential applications associated with this type of material [1,2]. MOFs present incomparable properties due to their crystalline nature, high thermal stability, tunable chemical functionality, and ultra-high porosity [3]. In addition, their inorganic–organic hybrid nature gives them physical and chemical properties of both components (optical, photonic, magnetic properties, among others). Moreover, due to their host–guest properties, MOFs have been widely studied for their application for gas and volatile organic compound (VOC) capture, storage, and separation [4,5], selective sensing [6,7], and heterogeneous catalysis [8,9,10,11], among others [12].

One interesting approach for the design of new coordination polymers is the use of metal chelating groups different from carboxylate, imidazole, and/or pyridine-based ligands commonly used to obtain porous MOFs [13]. In this sense, β-diketonates have recently started to be used as structural building blocks in coordination polymers [14]. Deprotonated β-diketonates act as metal chelating agents; hence, in order to obtain extended structures, other substituents are necessary to make them act as bridging ligands. This is the case of bis(β-diketonate) 1,1,2,2-tetraacetylethane (H_2_Tae) which can act as bischelating ligand bridging two metal centers. The examples of coordination polymers built up from only Tae as linker are scarce [15,16], but the combination of Tae with pyridinic bridging ligands seems to be a good strategy to enlarge the high-dimensional coordination polymers. However, only a 3D architecture based on M-Tae-pyridinic ligands has been reported: the three [Cu_2_(Tae)(4,4′-Bpy)_2_]^2+^ [(NO_3_)_2_]^2−^ (4,4′-Bpy= 4,4′-Dipyridyl) concomitant supramolecular isomers reported by Luisi et al. [17]. During the course of our research with the Cu−H_2_Tae−4,4′-Bpy system, we have obtained the 1D {Cu(NO_3_)(H_2_O)}(HTae)(4,4′-Bpy) coordination polymer [18]. The crystal structure consists of parallel and oblique {Cu(HTae)(4,4′-Bpy)} zig-zag metal–organic chains stacked along the [100] crystallographic direction. The coordination environment of the copper atoms is formed by two nitrogen atoms of two bridging 4,4′-Bpy and two oxygen atoms of one chelating HTae molecules in the equatorial plane. The octahedral coordination is completed by two more oxygen atoms corresponding to coordinated water molecules and nitrate anions in the axial positions. The occupation of these positions varies from one copper atom to another, with different combinations of water and nitrate molecules, giving rise to a commensurate super-structure. 

Copper acetylacetonates have previously been used as homogeneous catalysts for the synthesis of epoxides and aziridines [19,20,21,22]. However, the major drawbacks towards a sustainable chemistry of homogenous catalysts are their difficult separation from the reaction media and recycling, as well as their low chemical and thermal stability. In order to overcome these problems, the encapsulation of copper acetylacetonates within inorganic porous compounds and polymers has been proven as an efficient solution [23,24,25] to recover the catalyst, maintaining the catalytic activity of the copper acetylcetonate [26,27,28,29,30].

With the same aim of immobilizing copper acetylacetonate compounds in a heterogeneous catalyst matrix, our strategy is focused on the construction of coordination polymers with Cu(II)-bis(β-diketonate) as building blocks. The Cu(II)-bis(β-diketonate) fragments could retain the catalytic activity of the Cu acetylcetonate homogeneous catalyst, opening the possibility to be used as building blocks of coordination polymers, and, hence, to design new metal–organic heterogeneous catalysts. The {Cu(NO_3_)(H_2_O)}(HTae)(4,4′-Bpy) compound is one of the results achieved following this strategy. In this specific case, the bis(β-diketonate) ligand acts as a simple chelating ligand. Starting from this coordination polymer, and by means of the thermal removal of water molecules, we were able to obtain the anhydrous {Cu(NO_3_)}(HTae)(4,4′-Bpy) compound, containing copper coordinatively unsaturated centers. These open metal sites could act as Lewis acid active centers in several heterogeneous catalyzed reactions. Therefore, we have tested the anhydrous compound as a heterogeneous catalyst for Knoevenagel condensation reactions.

## 2. Results and Discussion

### 2.1. Crystal Structure Description

**Figure 1:**
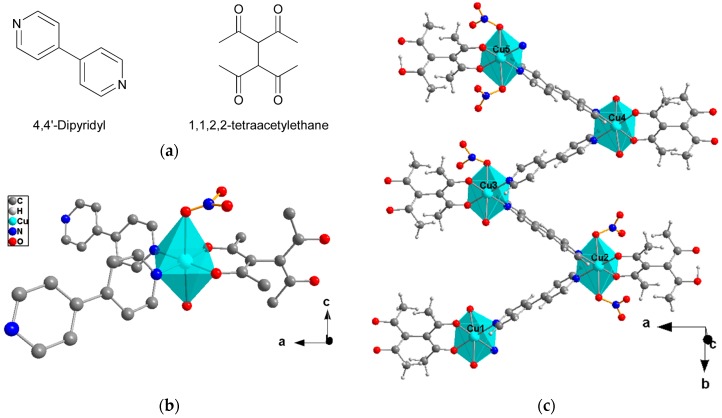
(**a**) Schematic representation of the ligands contained in {Cu(NO_3_)(H_2_O)}(HTae)(4,4′-Bpy) (**CuHTaeBpy_RT**); (**b**) One copper coordination environment of the **CuHTaeBpy_RT** crystal structure (hydrogen atoms were suppressed for clarity); (**c**) Cu−4,4′-Bpy−HTae metal–organic chains. Notice that the coordination environment of the copper(II) cations changes along the chain.

A detailed crystallographic study and structural description of the studied compound can be found in the recent work of Fernández de Luis et al. [18], but a brief description of the crystal structure is necessary for a better understanding of the activation process and catalytic properties of the material. {Cu(NO_3_)(H_2_O)}(HTae)(4,4′-Bpy) (hereafter, **CuHTaeBpy_RT**) is a one-dimensional coordination compound constructed from Cu−4,4′-Bpy zig-zag metal–organic chains running along the [011] and [0-11] crystallographic directions (Figure 1). While the 4,4′-Bpy ligand acts as a bridge between copper cations, the HTae molecule is partially deprotonated and acts as a simple chelating ligand, only linked to one copper cation of the metal–organic chain. Connectivity of the Cu(II) with the 4,4′-Bpy and HTae ligand complete the equatorial plane of its octahedral coordination environment. Coordinatively-bonded water molecules and nitrate anions are found in the axial positions of the octahedral (Figure 1a). In fact, there is a commensurate occupational coupled disorder of the nitrate and water molecules, giving rise to a five-fold order of the crystal structure along the c* axis (Figure 1b). As a consequence of the structure commensurability, three different coordination environments of copper cations are present in the crystal structure:

- Cu(II) atoms bonded to two coordinated water molecules.
- Cu(II) atoms bonded to one water molecule and one nitrate group.
- Cu(II) atoms connected to two nitrate anions.

The Cu-O_water_ and Cu-O_nitrate_ bond distances—which take values from 2.517(8) to 2.604(9) Å—are appreciably longer than those of the equatorial plane Cu-O and Cu-N bonds (ranging from 1.882(6) to 2.022(6) Å), due to the Jahn–Teller effect typical of Cu(II) cations. Even so, these long distances contribute effectively to the bond valence of the copper atoms. This commensurability of the coordination sphere also has an appraisable impact on the thermal behavior of the **CuHTaeBpy_RT** compound.

### 2.2. Thermal and Spectroscopic Characterization

The study of the thermal stability of **CuHTaeBpy_RT** is of great importance to the establishment of a proper activation temperature without reaching the collapse of the crystal framework. A detailed study of the thermal properties has been reported previously, but describing the most important features here is essential in order to understand the activation process, and to compare the catalyst before and after the catalytic processes.

**Figure 2:**
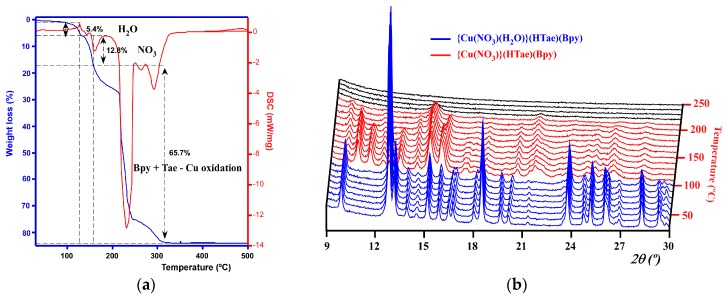
(**a**) Thermogravimetric and differential scanning calorimetry (DSC) analyses; (**b**) Thermodiffractometry.

Thermogravimetric and thermodiffractometric studies reveal four stages in the thermal response of {Cu(NO_3_)(H_2_O)}(HTae)(4,4′-Bpy) (Figure 2). The loss of the coordinated water molecules occurs between 100 °C and 160 °C, and gives rise to an irreversible structural transformation. There is a great loss of crystallinity during the process, as is deduced by the X-ray diffraction peak broadening. Hereafter, the anhydrous compound obtained above 100 °C will be denoted as **CuHTaeBpy_HT**, in contrast with the as-synthesized one, **CuHTaeBpy_RT**. Above 160 °C, nitrate groups are released from the crystal structure, causing the structure to collapse due to the charge imbalance. Once the crystal structure has collapsed, the calcination of the HTae and 4,4′-Bpy ligands takes place from 220 to 320 °C. Taking into account the above-described thermal behavior, the activation of the compound and the creation of copper coordinatively unsaturated centers can be reached by the activation of **CuHTaeBpy_RT** above 100 °C and below 160 °C in air conditions. This temperature could be reduced to 80 °C when using vacuum conditions.

**Figure 3:**
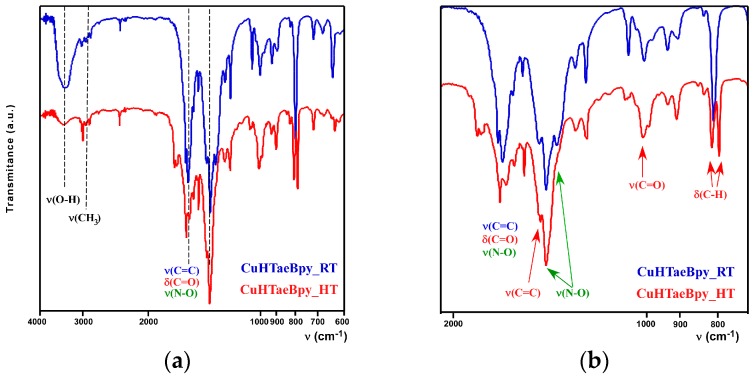
(**a**) IR spectra for the hydrated **CuHTaeBpy_RT** and anhydrous **CuHTaeBpy_HT** compounds; (**b**) Detailed range (2000–600 cm^−1^) of the IR spectra.

Spectroscopic studies of both the room temperature compound and the compound after dehydration at 120 °C for 30 min were carried out. The IR spectra for **CuHTaeBpy_RT** and **CuHTaeBpy_HT** show the characteristic absorption bands of the water molecules, nitrate groups, and 4,4′-Bpy and HTae ligands (Figure 3). The stretching vibration of the O-H bonds generates a broad absorption band centered at 3425 cm^−1^. This value goes along with previously reported values for H-bonded water molecules, in good agreement with the 2D hydrogen bonding network observed in the crystal structure. The multiple absorption maxima observed between 1650 and 1500 cm^−1^ have been tentatively assigned to the stretching vibrations of the C=C and C=N bonds belonging to the 4,4-Bpy bridging ligand, stretching vibration of the N-O related to the nitrate anions, and to the bending of C-O groups of the HTae ligand (Figure 3) [31,32,33]. Among the absorption maxima observed below 1500 cm^−1^ (Figure 3b), the most intense ones are related to the stretching vibrations of C=C belonging to the HTae ligand at 1400 cm^−1^, and N-O stretching vibrations of the nitrate anions at 1380 and 1336 cm^−1^. At 1010 cm^−1^, a medium-intensity band of the C=O bonds of the HTae ligand is observed. Finally, at 815 and 798 cm^−1^, bands related to the C-H bending vibrations appear.

After the structural transformation, the main absorption bands of the IR spectra are retained, so the structural blocks of the crystal structure are maintained. However, there are several changes in the **CuHTaeBpy_HT** spectrum. The loss of intensity in the absorption bands assigned to the vibration modes of the coordinated water molecules is in good agreement with the loss of coordinated water molecules. In addition to this, there are several slight position shifts and intensity variations of some vibration modes. These facts suggest that, despite the fact that structural building blocks are retained, the crystal structure is reorganized after the loss of coordinated water molecules.

### 2.3. Catalytic Activity Study

The activation process induces the structural transformation of the initial hydrated compound into the anhydrous phase (**CuHTaeBpy_HT**) containing copper unsaturated metal centers. The combination of temperature and vacuum reduces the transformation temperature at which the coordination water molecules are released to 80 °C. The catalytic activity of the activated material has been tested for Knoevenagel reactions [34,35].

#### 2.3.1. Knoevenagel Condensation

**Scheme 1:**
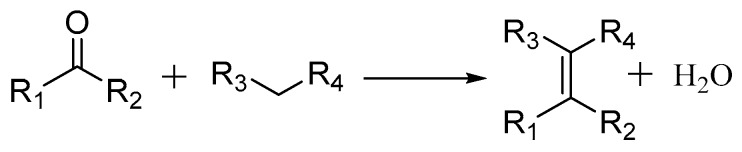
Knoevenagel condensation general scheme.

**Figure 4:**
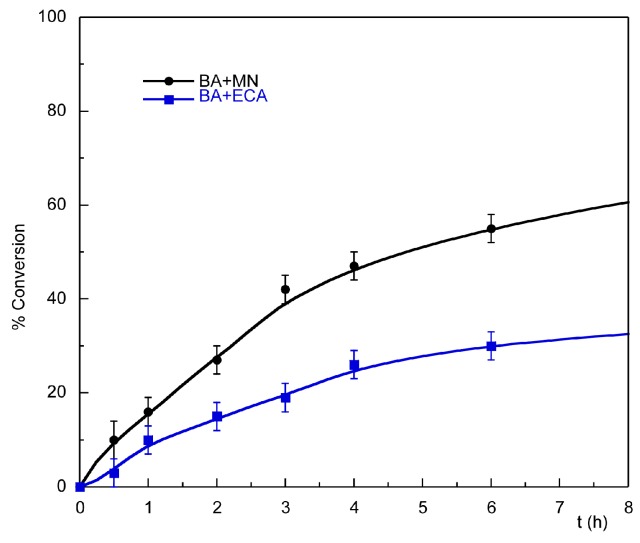
Kinetic profile of the Knoevenagel reactions of benzaldehyde (BA) with malononitrile (MN) and ethyl cyanoacetate (ECA). Reaction conditions: 60 °C, 3% catalyst, in toluene. Solid lines are guiding lines to help the visualization of the kinetic profiles.

Knoevenagel reactions (Scheme 1) were initially performed between benzaldehyde and malononitrile and ethyl cyanoacetate in the presence of 5% of catalyst (catalyst:substrate molar ratio). Taking into account the structural characteristics of the compound, the percentage of active cooper sites is 3% instead of 5%, since only 3 of the 5 copper(II) ions are coordinated to water molecules. This ratio (active sites:substrate molar ratio) is actually more accurate to describe the reaction conditions, and hereafter it will be described this way. At first, the reactions were performed at 100 °C, observing the catalyst dissolution and the consequent appearance of peaks corresponding to 4,4′-Bpy and Tae in the chromatograms. The temperature was decreased to 60 °C to avoid catalyst dissolution. As a consequence of the difference in the p*K*_a_ of malononitrile (p*K*_a_ ≈ 7) and ethyl cyanoacetate (p*K*_a_ ≈ 11), the yield after 24 h of reaction with malononitrile (84%) was higher than that with ethyl cyanoacetate (15%). The kinetic profiles of both reactions are shown in Figure 4.

In order to study the scope of the reaction, malononitrile was selected to react with various aldehydes and ketones (Table 1). The reaction with ketones is much more hindered, and only a little conversion is observed for cyclohexanone (entry No. 7, Table 1). The conversion for acetophenone and cyclopentanone is almost negligible, and the results are not shown in the table.

Regarding the reactions carried out with aromatic aldehydes, the introduction of substituents in the aromatic ring produces a slight decrease in the final reaction conversion (entries No. 3–5 in Table 1) due to the increase of steric hindrance. The conversion values are very similar, without the influence of donor–acceptor character from substituents on the aromatic ring. Linear heptanal (entry No. 6) reacts more rapidly than benzaldehyde, taking into account the conversion at 2 h, but the total conversion reached after 24 h is very similar.

After the reactions, the solid catalyst was recovered by centrifugation and washed with toluene and acetone. Once dried, it was characterized by powder X-ray diffraction and IR spectroscopy (KBr pellet). The results of this characterization will be described in Section 2.3.3.

#### 2.3.2. Recycling and Heterogeneity Tests

**Figure 5:**
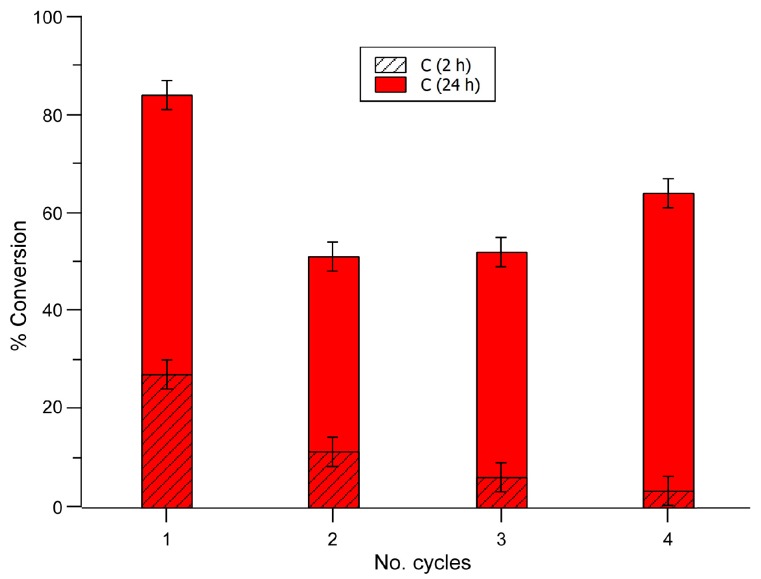
Recycling experiments for the Knoevenagel condensation of benzaldehyde and malononitrile (60 °C, 3 mol % catalyst, in toluene).

Recycling is the most interesting feature of heterogeneous catalysts. After letting **CuHTaeBpy_HT** react for 24 h, it was washed with toluene to remove the reagents and product, and the reactor was recharged with fresh reagents to start the reaction again. This process was repeated three times, taking aliquots at 2 and 24 h to evaluate the recyclability of the catalyst. The results (Figure 5) show a decrease in the conversion after 2 h along the cycles, indicating that the reactivity of the catalyst decreases. However, the total conversion after 24 h does not show the same decreasing pattern. This value, a decrease between the first and the second reaction cycle, is maintained between the second and the third, and slightly increases in the fourth reaction cycle. A tentative explanation of this behavior will be described in Section 2.3.3.

The catalyst recovered after the fourth cycle was characterized by powder X-ray diffraction and IR spectroscopy, and the results will be described in Section 2.3.3.

**Figure 6:**
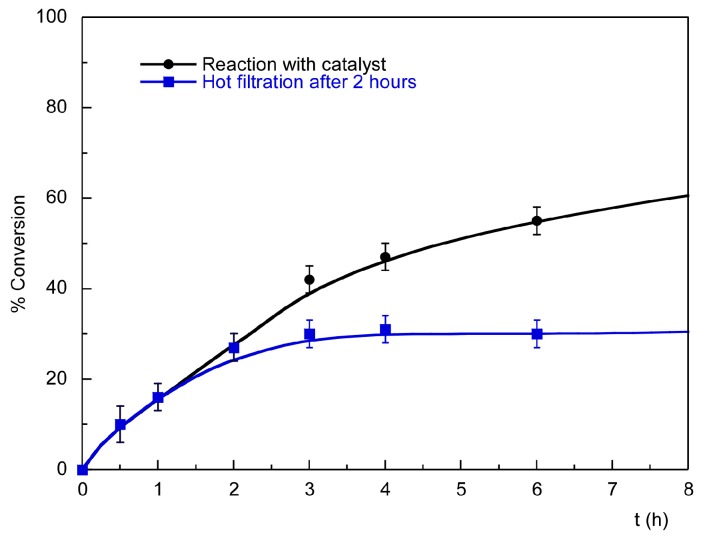
Comparison of the kinetic profiles of the reactions with catalyst (black) and after hot filtration at 2 h (blue). Solid lines are guiding line to help the visualization of the kinetic profiles.

In order to confirm the heterogeneous nature of the catalytic process, a hot filtration test was performed. The reaction was carried out under the typical conditions, and the mixture was filtered after 2 h. The liquid was allowed to react for 6 h. The reaction conversion increased slightly after the filtration (from 27% to 30%), but it was maintained at 30% until 6 h (Figure 6). This result indicates that there were no leaching processes during the reaction.

#### 2.3.3. Catalyst Characterization

X-ray diffraction and IR spectroscopy were used to study the catalyst after the activation process and catalytic reactions. The **CuHTaeBpy_HT** phase was much less crystalline than **CuHTaeBpy_RT**, so the catalyst samples recovered after the reaction are also poorly crystalline (Figures S1–S3). The crystallinity decreases with the reutilization of the catalyst, as can be deduced from Figure S3. In addition to the loss of crystallinity, the position shifting of some reflections suggests that the structure of the anhydrous compound has changed after the reaction. However, further studies are necessary to find out the possible structural and chemical changes occurring during the catalytic reaction.

**Figure 7:**
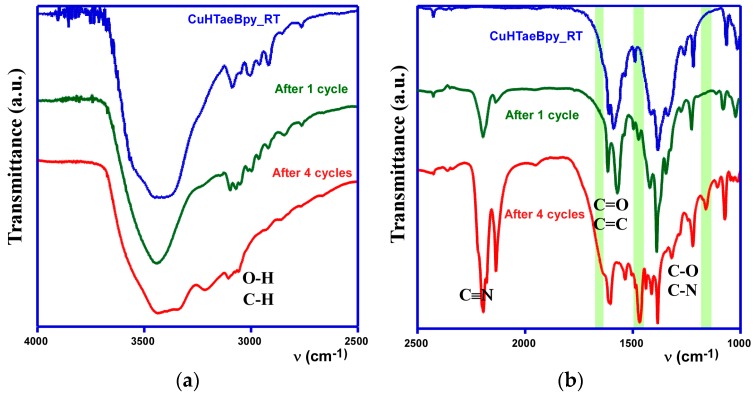
Details of the IR spectra of the **CuHTaeBpy_RT** preactivated catalyst, the catalyst recovered after 1 cycle, and after 4 cycles of reaction: (**a**) between 4000 and 2500 cm^−1^ and (**b**) in the 2500–1000 cm^−1^ range. Green boxes are used to point out the extra bands observed for the catalyst recovered after 4 cycles.

Infrared spectra are very similar to the spectrum of **CuHTaeBpy_HT**; the main absorption maxima—and hence, the vibration modes of the functional groups present in the original compound—are retained. Nevertheless, slight changes below 2000 cm^−1^, and the presence of two intense additional bands located at 2200 and 2120 cm^−1^ were observed (Figure 7 and Figures S4 and S5). These bands could be related with the presence of nitrile groups chemisorbed into the catalyst during the reactions. The nitrile groups interact with the coordinatively unsaturated copper centers, facilitating the removal of one hydrogen atom from the active methylene of malononitrile, a reaction mechanism previously proposed by Položij et al. [36]. The mechanism followed in the reaction of benzaldehyde and ethyl cyanoacetate should be different, because the bands corresponding to the nitrile groups are not observed in the IR spectrum (Figure S4).

In the IR spectrum of the catalyst recovered after four cycles, additional absorption bands are observed at 1158, 1466, and 1637 cm^−1^ (green boxes in Figure 7a). These bands can be assigned to benzaldehyde, which could have gotten trapped into the catalyst along the successive cycles. This could explain the changes observed in the conversion values when recycling the catalyst. The amount of benzaldehyde trapped in the catalyst increases with the number of cycles, hindering the approaching of the malononitrile to the active centers, and causing a decrease in the conversion rates.

## 3. Materials and Methods

### 3.1. Synthesis

Commercially available reagent grade chemicals were purchased from Sigma-Aldrich, and used without further purification: 4,4′-Dipyridyl (4,4′-Bpy), 1,1,2,2-tetraacetylethane (H_2_Tae), Cu(NO_3_)_2_·H_2_O, and ethanol.

**CuHTaeBpy_RT** coordination polymer was crystallized by the evaporation method. H_2_Tae ligand (0.040 g, 0.2 mmol) and copper nitrate hydrate (0.038 g, 0.2 mmol) were dissolved at 80 °C in 36 mL of ethanol. Once the complete dissolution of the H_2_Tae reagent is reached, the temperature was lowered to 35 °C, and 0.080 g of 4,4′-Bpy (0.5 mmol) were added. Immediately after adding the 4,4′-Bpy, the color of the solution changed from a pale to a dark green. Single-crystals were obtained after three days of slow evaporation. The samples were washed thoroughly with ethanol, and dried at room temperature.

### 3.2. Characterization

Samples were characterized routinely by powder X-ray diffraction (PXRD), infrared spectroscopy (IR), and elemental analysis (Exp.: H 4.66(7)%, C 48.6(6)%, and N 8.61(5)%, Theor.: H 5.05%, C 48.14%, and N 8.42%) before their use in catalytic studies. PXRD data were recorded in a Bruker D8 Advance Vårio diffractometer (Bruker, Billerica, MA, USA) Cu*Kα_1_* radiation, 2*θ* range = 5–70 °, step size = 0.015 °, exposure time = 10 s per step, at room temperature. Rietveld refinements with the average structural model and commensurate structural model were carried out. Despite the fact that there are no significant differences between the obtained agreement factors, the super-structural model was able to fit some low intensity satellite reflections not taken into account by the average model. This is important in order to avoid the association of these low intense peaks with impurities (Figure S6). The infrared spectra were recorded on a Jasco FT/IR-6100 spectrometer with pressed KBr pellets (400–4000 cm^−1^).

### 3.3. Themal Studies

Thermal analyses were performed in air atmosphere, up to 500 °C, with a heating rate of 5 °C/min on a Netzsch Sta Simultaneous DSC-TGA (Erich NETZSCH GmbH & Co. Holding KG, Selb, Germany). The temperature-dependent PXRD in air atmosphere was carried out on a Bruker D8 Advance Vantec diffractometer (Cu*K*α radiation), equipped with a variable-temperature stage HTK2000 for the measurements performed in the 30–400 °C temperature range (2*θ* range = 9–30 °, step size = 0.01 °, exposure time = 0.5 s per step). After understanding the thermal response of the material, the proper design of the activation conditions for the catalysis experiments can be proposed.

### 3.4. Catalytic Studies

Before the catalytic tests, the compound was dried at 80 °C in a vacuum oven in order to remove the water molecules present in the structure, obtaining the **CuHTaeBpy_HT** phase. The reaction conditions were established based on the reaction between benzaldehyde and malononitrile. Once the conditions were set, the reaction was made with benzaldehyde and ethyl cyanoacetate, and with different aldehydes or ketones: *p*-tolualdehyde, *p*-fluorobenzaldehyde, *p*-metoxybenzaldehyde, heptanal, acetophenone, cyclopentanone, and cyclohexanone. For a typical reaction, 10 mg (0.02 mmol) of catalyst were placed in a 2 mL vial with 0.5 mL of toluene anhydrous, 0.4 mmol of the substrate, 10 μL of dodecane as internal standard for GC-MS, and 0.6 mmol of malononitrile or ethyl cyanoacetate. Aliquots of the reaction media were taken at specific reaction times. The aliquots were analyzed by GC-MS (Agilent Technologies, Santa Clara, CA, USA) to determine the reaction progress.

A hot filtration test was performed for the reaction of benzaldehyde and malononitrile. The reactor was charged as usual and, after 2 h, the liquid was separated from the solid catalyst by filtration. The liquid was allowed to react, and aliquot samples were analyzed by GC-MS.

Reusing tests were also carried out for the reaction with benzaldehyde. After each cycle, the catalyst was recovered by centrifugation, washed with toluene three times, and dried at air conditions. Then, the reactor was charged again with the recovered catalyst and fresh reactants. This process was repeated four times. After all the reactions, the catalyst was recovered and analyzed by PXRD and IR spectroscopy (Jasco, Easton, MD, USA).

## 4. Conclusions

The use of bis(β-diketonate) 1,1,2,2-tetraacetylethane has yielded a new 1D coordination polymer with copper atoms in an octahedral coordination environment with the axial positions occupied by water molecules and nitrate anions. The water molecules can be removed from the structure with a thermal treatment, giving rise to unsaturated copper centers which are active sites for catalytic reactions which require Lewis acid sites. The catalyst is active for the Knoevenagel condensation of aldehydes with malononitrile at 60 °C. The tests have demonstrated that in these conditions, the catalyst is heterogeneous and can be reused for at least for four cycles, suffering a poisoning effect along the successive cycles by the reagents, which causes a decrease in the conversion rates to stable values between 60% and 65%.

## Figures and Tables

**Table 1:** Scope of **CuHTaeBpy_HT**-catalyzed Knoevenagel condensations ^1^.

Entry	Substrate	Donor	Conversion [%] (after 2 h)	Conversion [%] (after 24 h)
1	Benzaldehyde	Malononitrile	27	84
2	Benzaldehyde	Ethyl cyanoacetate	15	40
3	*p*-Tolualdehyde	Malononitrile	36	68
4	*p*-Metoxybenzaldehyde	Malononitrile	22	75
5	*p*-Fluorobenzaldehyde	Malononitrile	28	74
6	Heptanal	Malononitrile	51	89
7	Cyclohexanone	Malononitrile	2	11

^1^ Reaction conditions: 60 °C, 3% catalyst, in toluene.

## Supplementary Materials

Supplementary materials can be accessed at: http://www.mdpi.com/1420-3049/21/12/1651/s1.
