# Supplementary material for: Catalytic Performance of a New 1D Cu(II) Coordination Polymer {Cu(NO3)(H2O)}(HTae)(4,4′-Bpy) for Knoevenagel Condensation
Source: Molecules. 2016 Dec 1;21(12):1651. doi: 10.3390/molecules21121651 (PMC6272964; doi:10.3390/molecules21121651)
Supplement: Supplementary file 1 [file molecules-21-01651-s001.pdf]

# Supplementary Materials: Catalytic Performance of a New 1D Cu(II) Coordination Polymer {$Cu(NO_3)(H_2O)$}(HTae)(4,4′-Bpy) for Knoevenagel Condensation

**Edurne S. Larrea, Roberto Fernández de Luis and María I. Arriortua**

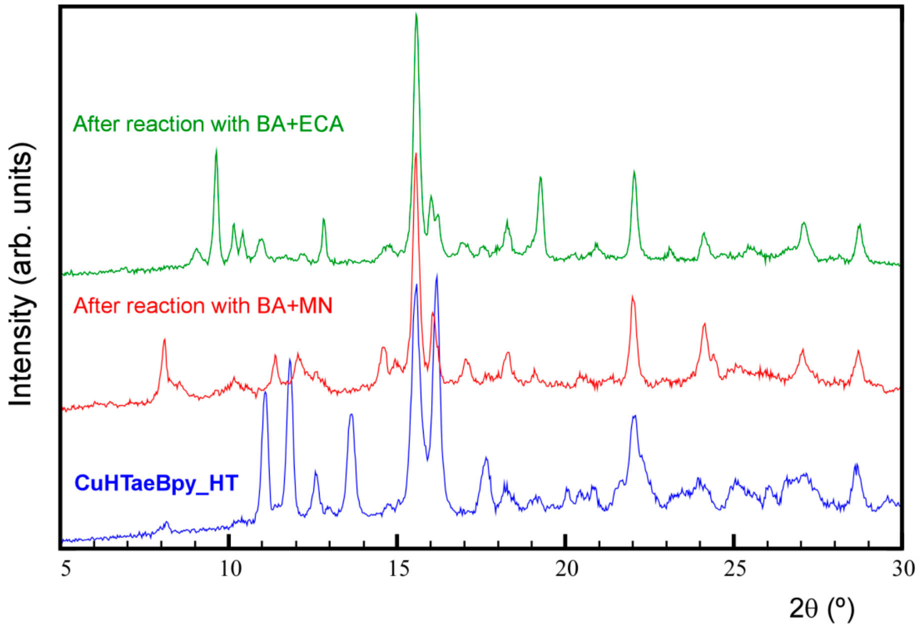


**Figure S1.** X-ray diffractograms of the activated **CuHTaeBpy_HT** catalyst before the reaction, after reacting with benzaldehyde (BA) and malononitrile (MN), and after reacting with benzaldehyde (BA) and ethyl cyanoacetate (ECA).

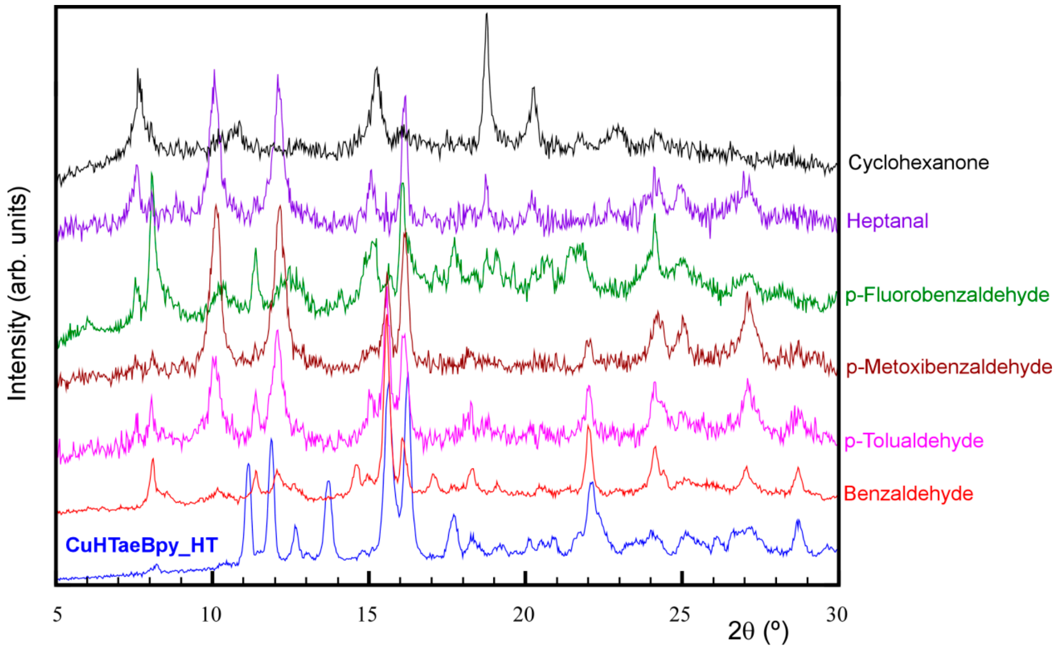


**Figure S2.** X-ray diffraction patterns of the activated catalyst, **CuHTaeBpy_HT**, and after reacting with various substrates and MN.

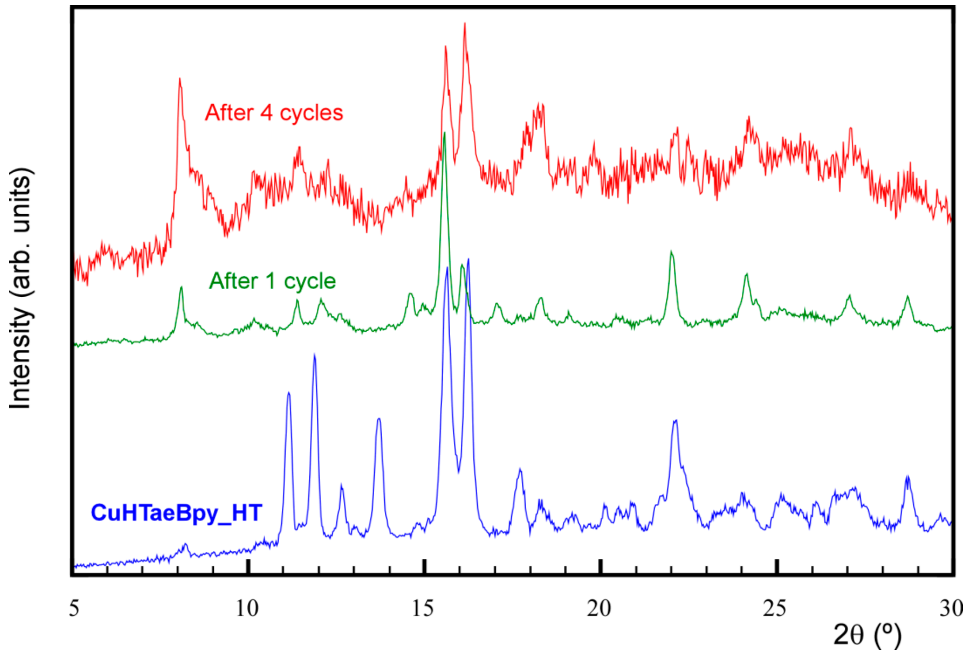


**Figure S3.** X-ray diffraction patterns of the activated catalyst, **CuHTaeBpy_HT**, and after 1 cycle of reaction and after 4 cycles of reaction of BA and MN.

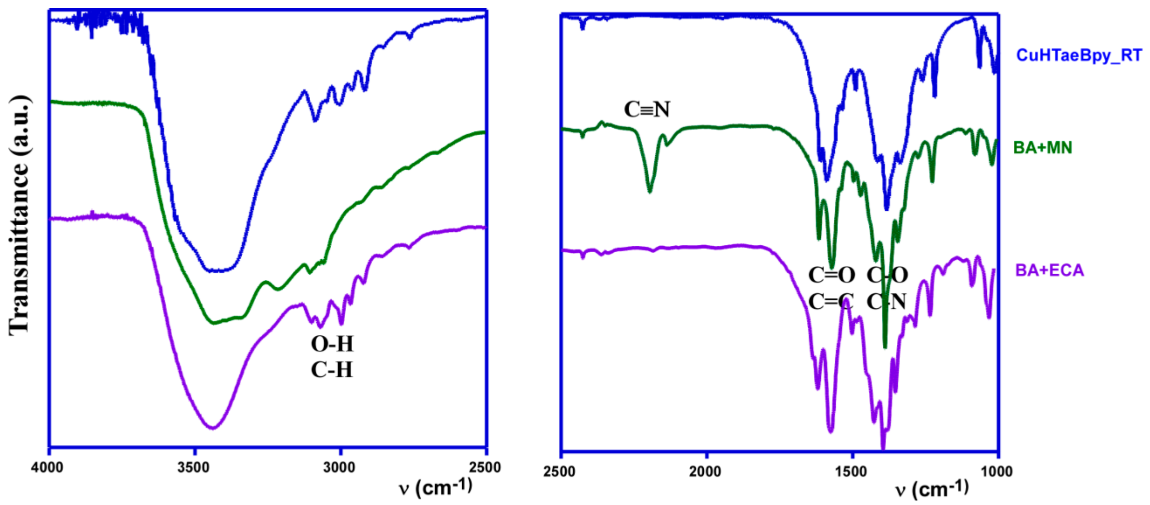


**Figure S4.** IR spectra of the **CuHTaeBpy_RT** preactivated catalyst, the catalyst after reacting with benzaldehyde (BA) and malononitrile (MN), and after reacting with benzaldehyde (BA) and ethyl cyanoacetate (ECA).

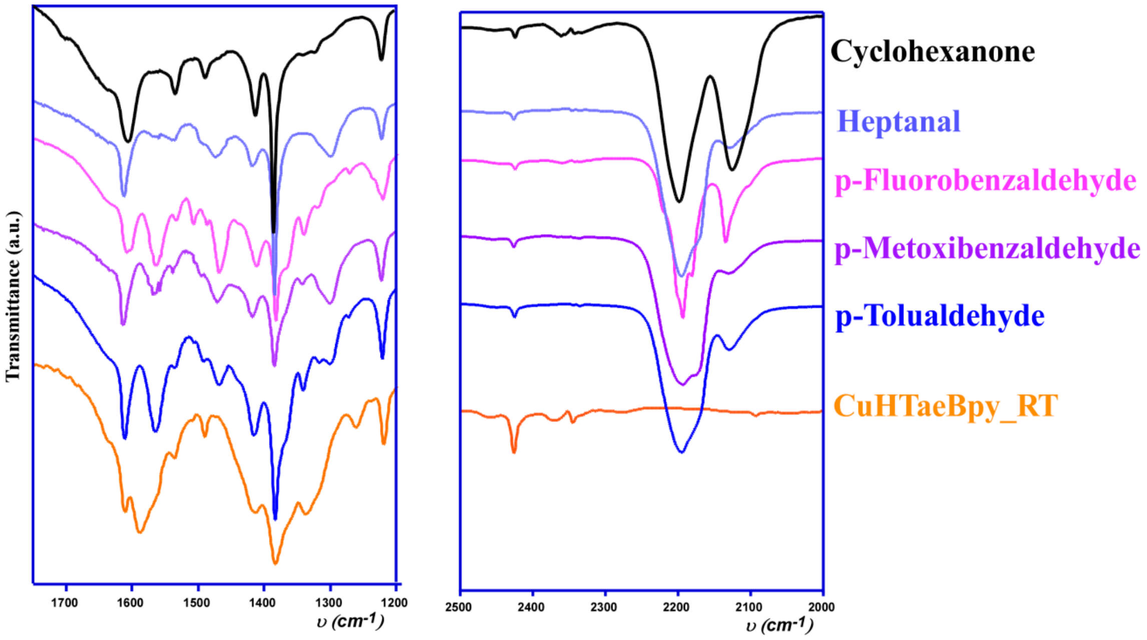


**Figure S5.** IR spectra of the **CuHTaeBpy_RT** preactivated catalyst, and the catalyst after reacting with various substrates and malononitrile.

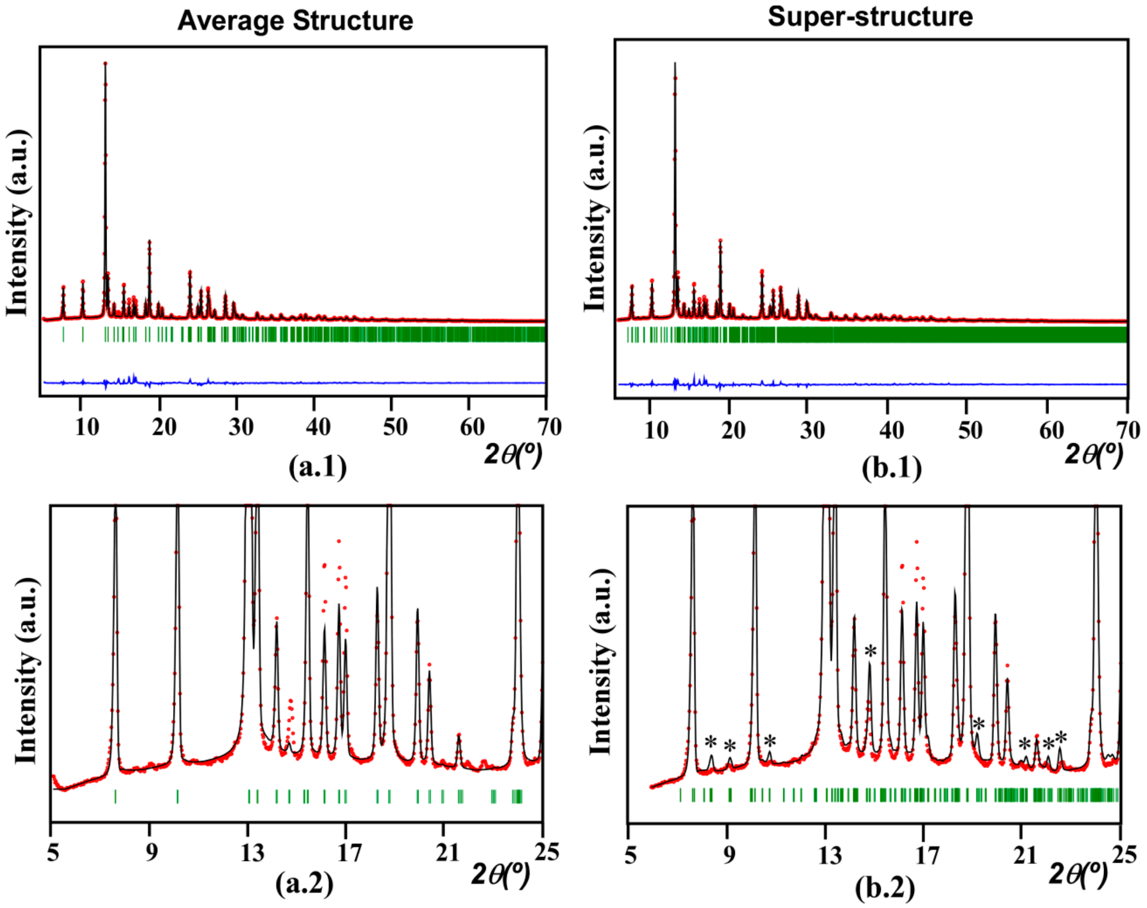


**Figure S6.** Rietveld refinements of the {Cu(NO3)(H2O)}(HTae)(4,4′-Bpy) with the average structural model (**a.1**) and commensurate structural model (**b.1**). Details of the refinement of the satellite peaks due to the super-structure with the average structural model (**a.2**) and with the commensurate structural model (**b.2**).
